# 3-(1-Adamant­yl)-4-amino-1-(2-benzoyl-1-phenyl­eth­yl)-1*H*-1,2,4-triazol-5(4*H*)-thione

**DOI:** 10.1107/S1600536812015929

**Published:** 2012-04-18

**Authors:** Siham Lahsasni, Ali A. El-Emam, Nasser R. El-Brollosy, Ching Kheng Quah, Hoong-Kun Fun

**Affiliations:** a Department of Chemistry, College of Sciences, King Saud University, Riyadh, Saudi Arabia; b Department of Pharmaceutical Chemistry, College of Pharmacy, King Saud University, Riyadh 11451, Saudi Arabia; c X-ray Crystallography Unit, School of Physics, Universiti Sains Malaysia, 11800 USM, Penang, Malaysia

## Abstract

In the title compound, C_27_H_30_N_4_OS, the 3-(adamantan-1-yl)-4-amino-1*H*-1,2,4-triazole-5(4*H*)-thione unit and the O atom are each disordered over two sets of sites with refined site-occupancies of 0.7630 (13) and 0.2370 (13). The 1,2,4-triazole ring of the major component forms dihedral angles of 62.61 (17) and 61.93 (16)° with the benzene rings, while that of the minor component makes corresponding angles of 86.3 (4) and 79.1 (4)°. The dihedral angle between the benzene rings is 39.21 (16)°. The mol­ecular structure is stabilized by an intra­molecular C—H⋯N hydrogen bond, which generates an *S*(6) ring motif. In the crystal, mol­ecules are linked into inversion dimers by pairs of N—H⋯S hydrogen bonds.

## Related literature

For the biological activity of adamantane derivatives, see: Vernier *et al.* (1969 ▶); Kadi *et al.* (2007 ▶, 2010 ▶); Al-Abdullah *et al.* (2007 ▶); El-Emam *et al.* (2004 ▶). For related adamantyl-1,2,4-triazole structures, see: Al-Abdullah *et al.* (2012 ▶); Almutairi *et al.* (2012 ▶); Al-Tamimi *et al.* (2010 ▶). For related amino-1,2,4-triazole structures, see: Song *et al.* (2011 ▶); Gao *et al.* (2011 ▶); Wang *et al.* (2011 ▶). For standard bond-length data, see: Allen *et al.* (1987 ▶). For hydrogen-bond motifs, see: Bernstein *et al.* (1995 ▶).
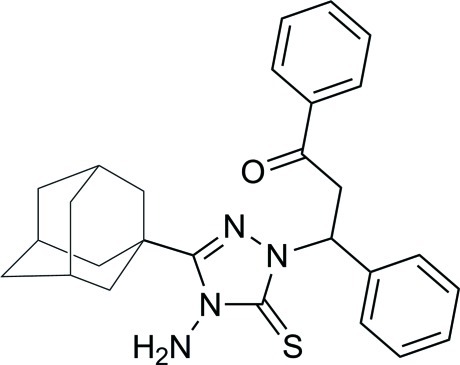


## Experimental

### 

#### Crystal data

C_27_H_30_N_4_OS
*M* *_r_* = 458.61
Monoclinic, 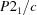

*a* = 11.9409 (3) Å
*b* = 9.5478 (3) Å
*c* = 22.0034 (6) Å
β = 103.610 (2)°
*V* = 2438.15 (12) Å^3^
*Z* = 4
Cu *K*α radiation
μ = 1.38 mm^−1^
*T* = 296 K
0.98 × 0.66 × 0.33 mm

#### Data collection

Bruker SMART APEXII CCD area-detector diffractometer
Absorption correction: multi-scan (*SADABS*; Bruker, 2009 ▶) *T*_min_ = 0.345, *T*_max_ = 0.662
18569 measured reflections
4512 independent reflections
3341 reflections with *I* > 2σ(*I*)
*R*_int_ = 0.041

#### Refinement

*R*[*F* ^2^ > 2σ(*F* ^2^)] = 0.057
*wR*(*F* ^2^) = 0.140
*S* = 1.04
4512 reflections
371 parameters
30 restraints
H-atom parameters constrained
Δρ_max_ = 0.17 e Å^−3^
Δρ_min_ = −0.18 e Å^−3^

### 

Data collection: *APEX2* (Bruker, 2009 ▶); cell refinement: *SAINT* (Bruker, 2009 ▶); data reduction: *SAINT*; program(s) used to solve structure: *SHELXTL* (Sheldrick, 2008 ▶); program(s) used to refine structure: *SHELXTL*; molecular graphics: *SHELXTL*; software used to prepare material for publication: *SHELXTL* and *PLATON* (Spek, 2009 ▶).

## Supplementary Material

Crystal structure: contains datablock(s) global, I. DOI: 10.1107/S1600536812015929/is5114sup1.cif

Structure factors: contains datablock(s) I. DOI: 10.1107/S1600536812015929/is5114Isup2.hkl

Supplementary material file. DOI: 10.1107/S1600536812015929/is5114Isup3.cml

Additional supplementary materials:  crystallographic information; 3D view; checkCIF report

## Figures and Tables

**Table 1:** Hydrogen-bond geometry (Å, °)

*D*—H⋯*A*	*D*—H	H⋯*A*	*D*⋯*A*	*D*—H⋯*A*
N4—H1*N*4⋯S1^i^	0.90	2.60	3.475 (3)	166
C4—H4*B*⋯N4	0.97	2.53	3.177 (4)	124

Symmetry code: (i) 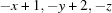.

**Fig. 1.**
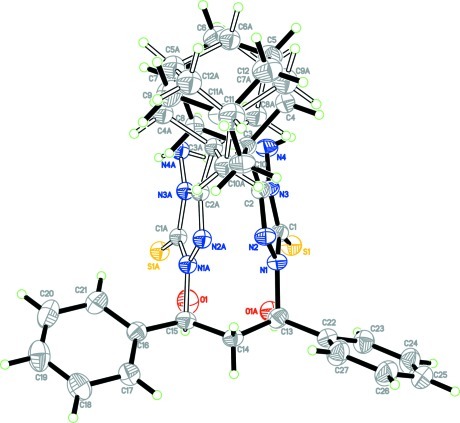
The molecular structure of the title compound showing 30% probability displacement ellipsoids for non-H atoms. All disorder components are shown.

**Fig. 2.**
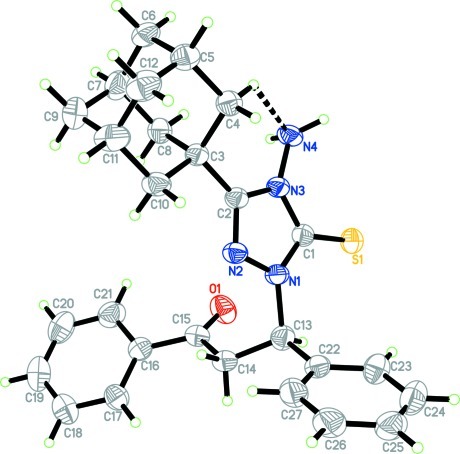
The molecular structure of the major component of the title compound showing 30% probability displacement ellipsoids for non-H atoms. The intramolecular hydrogen bond is shown as a dashed line.

**Fig. 3.**
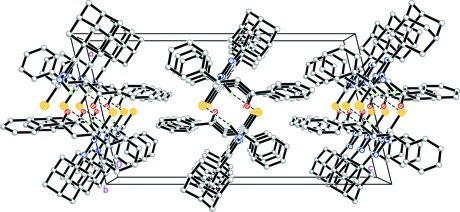
Part of the crystal structure of the title compound, viewed along the b axis. H atoms not involved in hydrogen bonds (dashed lines) have been omitted for clarity. Only the major disorder component is shown.

## supplementary crystallographic information

### Comment

Derivatives of adamantane have long been known for their diverse biological activities including antiviral activity against the influenza (Vernier *et al.*, 1969) and HIV viruses (El-Emam *et al.*, 2004). Moreover, adamantane derivative were recently reported to exhibit marked antibacterial activity (Kadi *et al.*, 2007, 2010). In continuation to our interest in the reactions of amino-1,2,4-triazoles (Al-Abdullah *et al.*, 2007), we report herein the synthesis and structure of the title compound (I) as potential chemotherapeutic agent.

In the title molecule, Fig. 1, the 3-(adamantan-1-yl)-4-amino-1*H*-1,2,4-triazole-5(4*H*)-thione moiety and an oxygen atom are disordered over two positions with refined site-occupancies of 0.763 (1):0.237 (1). The mean plane of major component of 1,2,4-triazole ring (N1-N3/C1/C2, r.m.s deviation = 0.003 Å) forms dihedral angles of 62.61 (17) and 61.93 (16)° with the two benzene rings (C16–C21 and C22–C27). The corresponding angles for minor component of 1,2,4-triazole ring (r.m.s deviation = 0.010 Å) are 86.3 (4) and 79.1 (4)°. The dihedral angle between the two benzene rings is 39.21 (16)°. Bond lengths (Allen *et al.*, 1987) and angles are within normal ranges. The molecular structure is stabilized by an intramolecular C4—H4B···N4 hydrogen bond (Table 1), which generates an *S*(6) ring motif (Fig. 2; Bernstein *et al.*, 1995).

In the crystal (Fig. 3), molecules are linked into inversion dimers by pairs of N4—H1N4···S1 hydrogen bonds (Table 1).

### Experimental

A mixture of 3-(1-adamantyl)-4-amino-4*H*-1,2,4-triazole-5-thiol (2.5 g, 0.01 mol) and (*E*)-1-(4-phenyl)-3-phenylprop-2-en-1-one (2.08 g, 0.01 mol), in ethanol (15 ml), was heated under reflux for 10 h and the solvent was then distilled off *in vacuo*. The resulted residue was eluted from silica gel column using hexane:ethyl acetate (4:1) to yield 1.01 g (22%) of the title compound (I) as pale yellow powder. *M.p.* 191-193°C. Crystals of (I) suitable for single crystal X-ray analysis were grown by slow evaporation of a solution in chloroform-ethanol (1:1) at room temperature. ^1^H NMR (CDCl_3_, 500.13 MHz): δ 1.66 (s, 6H, Adamantane-H), 1.95 (s, 9H, Adamantane-H), 3.45-3.49 (m, 1H^a^, CH_2_CO), 4.23-4.29 (m, 1H^b^, CH_2_CO), 4.51 (s, 2H, NH_2_), 6.48-6.51 (m, 1H, CH), 7.18-7.27 (m, 3H, Ar-H), 7.36-7.48 (m, 5H, Ar-H), 7.88-7.90 (m, 2H, Ar-H). ^13^C NMR (CDCl_3_, 125.76 MHz): δ 27.84, 35.0, 36.42, 38.41 (Adamantane-C), 42.75 (CH), 58.09 (CH_2_), 127.54, 128.14, 128.24, 128.60, 128.71, 133.30, 136.66, 138.71 (Ar-C), 156.25 (Triazole C-3), 167.55 (C=S), 196.09 (C=O).

### Refinement

The 3-(adamantan-1-yl)-4-amino-1*H*-1,2,4-triazole-5(4*H*)-thione moiety and the oxygen atom are disordered over two positions with refined site-occupancies of 0.763 (1) : 0.237 (1). All minor disordered components were refined isotropically. All hydrogen atoms were positioned geometrically (N—H = 0.8979 Å and C—H = 0.93–0.98 Å) and were refined using a riding model, with *U*_iso_(H) = 1.2 *U*_eq_(N,C). The adamantyl (C3–C12) moiety was subjected to similarity restraints.

### Crystal data

**Table:**

C_27_H_30_N_4_OS	*F*(000) = 976
*M**_r_* = 458.61	*D*_x_ = 1.249 Mg m^−^^3^
Monoclinic, *P*2_1_/*c*	Cu *K*α radiation, λ = 1.54178 Å
Hall symbol: -P 2ybc	Cell parameters from 3061 reflections
*a* = 11.9409 (3) Å	θ = 3.8–67.9°
*b* = 9.5478 (3) Å	µ = 1.38 mm^−^^1^
*c* = 22.0034 (6) Å	*T* = 296 K
β = 103.610 (2)°	Plate, colourless
*V* = 2438.15 (12) Å^3^	0.98 × 0.66 × 0.33 mm
*Z* = 4	

### Data collection

**Table:**

Bruker SMART APEXII CCD area-detector diffractometer	4512 independent reflections
Radiation source: fine-focus sealed tube	3341 reflections with *I* > 2σ(*I*)
Graphite monochromator	*R*_int_ = 0.041
φ and ω scans	θ_max_ = 69.6°, θ_min_ = 3.8°
Absorption correction: multi-scan (*SADABS*; Bruker, 2009)	*h* = −14→13
*T*_min_ = 0.345, *T*_max_ = 0.662	*k* = −11→11
18569 measured reflections	*l* = −26→26

### Refinement

**Table:**

Refinement on *F*^2^	Primary atom site location: structure-invariant direct methods
Least-squares matrix: full	Secondary atom site location: difference Fourier map
*R*[*F*^2^ > 2σ(*F*^2^)] = 0.057	Hydrogen site location: inferred from neighbouring sites
*wR*(*F*^2^) = 0.140	H-atom parameters constrained
*S* = 1.04	*w* = 1/[σ^2^(*F*_o_^2^) + (0.0357*P*)^2^ + 1.3343*P*] where *P* = (*F*_o_^2^ + 2*F*_c_^2^)/3
4512 reflections	(Δ/σ)_max_ = 0.001
371 parameters	Δρ_max_ = 0.17 e Å^−^^3^
30 restraints	Δρ_min_ = −0.18 e Å^−^^3^

### Special details

**Table:**

Geometry. All esds (except the esd in the dihedral angle between two l.s. planes) are estimated using the full covariance matrix. The cell esds are taken into account individually in the estimation of esds in distances, angles and torsion angles; correlations between esds in cell parameters are only used when they are defined by crystal symmetry. An approximate (isotropic) treatment of cell esds is used for estimating esds involving l.s. planes.
Refinement. Refinement of F^2^ against ALL reflections. The weighted R-factor wR and goodness of fit S are based on F^2^, conventional R-factors R are based on F, with F set to zero for negative F^2^. The threshold expression of F^2^ > 2sigma(F^2^) is used only for calculating R-factors(gt) etc. and is not relevant to the choice of reflections for refinement. R-factors based on F^2^ are statistically about twice as large as those based on F, and R- factors based on ALL data will be even larger.

### Fractional atomic coordinates and isotropic or equivalent isotropic displacement parameters (Å^2^)

**Table:**

	*x*	*y*	*z*	*U*_iso_*/*U*_eq_	Occ. (<1)
S1	0.52127 (8)	0.89181 (9)	−0.08864 (4)	0.0666 (3)	0.7630 (13)
O1	0.5192 (2)	0.6892 (3)	0.05575 (12)	0.0788 (7)	0.7630 (13)
N1	0.7014 (2)	0.7645 (2)	−0.00707 (12)	0.0514 (6)	0.7630 (13)
N2	0.7971 (2)	0.7977 (2)	0.03974 (12)	0.0524 (6)	0.7630 (13)
N3	0.7028 (2)	0.9862 (2)	0.00087 (11)	0.0506 (6)	0.7630 (13)
N4	0.6719 (2)	1.1284 (2)	−0.00704 (13)	0.0632 (7)	0.7630 (13)
H1N4	0.6119	1.1217	0.0110	0.076*	0.7630 (13)
H2N4	0.6665	1.1741	−0.0433	0.076*	0.7630 (13)
C1	0.6410 (3)	0.8782 (3)	−0.03184 (14)	0.0517 (7)	0.7630 (13)
C2	0.7961 (2)	0.9338 (3)	0.04378 (14)	0.0485 (7)	0.7630 (13)
C3	0.8827 (3)	1.0177 (3)	0.08935 (15)	0.0498 (7)	0.7630 (13)
C4	0.9397 (3)	1.1307 (3)	0.05715 (15)	0.0600 (8)	0.7630 (13)
H4A	0.9763	1.0871	0.0270	0.072*	0.7630 (13)
H4B	0.8813	1.1948	0.0348	0.072*	0.7630 (13)
C5	1.0292 (4)	1.2118 (4)	0.1049 (2)	0.0706 (11)	0.7630 (13)
H5A	1.0648	1.2828	0.0834	0.085*	0.7630 (13)
C6	0.9684 (9)	1.2830 (7)	0.1497 (5)	0.089 (4)	0.7630 (13)
H6A	0.9094	1.3453	0.1266	0.107*	0.7630 (13)
H6B	1.0234	1.3387	0.1795	0.107*	0.7630 (13)
C7	0.9138 (4)	1.1753 (4)	0.18449 (18)	0.0736 (10)	0.7630 (13)
H7A	0.8757	1.2225	0.2137	0.088*	0.7630 (13)
C8	0.8246 (3)	1.0891 (4)	0.13665 (16)	0.0619 (8)	0.7630 (13)
H8A	0.7905	1.0185	0.1584	0.074*	0.7630 (13)
H8B	0.7635	1.1503	0.1147	0.074*	0.7630 (13)
C9	1.0063 (5)	1.0770 (5)	0.22011 (19)	0.0914 (13)	0.7630 (13)
H9A	0.9717	1.0077	0.2423	0.110*	0.7630 (13)
H9B	1.0625	1.1296	0.2506	0.110*	0.7630 (13)
C10	0.9749 (5)	0.9195 (4)	0.1271 (4)	0.0670 (19)	0.7630 (13)
H10A	1.0118	0.8690	0.0989	0.080*	0.7630 (13)
H10B	0.9389	0.8516	0.1492	0.080*	0.7630 (13)
C11	1.0660 (5)	1.0035 (5)	0.1744 (4)	0.0763 (16)	0.7630 (13)
H11A	1.1256	0.9399	0.1973	0.092*	0.7630 (13)
C12	1.1208 (3)	1.1131 (5)	0.1397 (2)	0.0857 (12)	0.7630 (13)
H12A	1.1788	1.1653	0.1693	0.103*	0.7630 (13)
H12B	1.1581	1.0670	0.1104	0.103*	0.7630 (13)
S1A	0.4172 (2)	0.8775 (3)	0.06445 (13)	0.0634 (8)*	0.2370 (13)
O1A	0.5678 (6)	0.6617 (8)	−0.0404 (3)	0.0661 (19)*	0.2370 (13)
N1A	0.6323 (6)	0.7602 (8)	0.0891 (4)	0.0496 (18)*	0.2370 (13)
N2A	0.7482 (6)	0.7939 (8)	0.1048 (4)	0.0496 (18)*	0.2370 (13)
N3A	0.6374 (6)	0.9813 (8)	0.0936 (3)	0.0494 (18)*	0.2370 (13)
N4A	0.6033 (7)	1.1229 (8)	0.0904 (4)	0.058 (2)*	0.2370 (13)
H3N4	0.5613	1.1566	0.0540	0.069*	0.2370 (13)
H4N4	0.5394	1.1375	0.1043	0.069*	0.2370 (13)
C1A	0.5601 (8)	0.8693 (10)	0.0806 (4)	0.050 (2)*	0.2370 (13)
C2A	0.7469 (8)	0.9328 (9)	0.1069 (4)	0.049 (2)*	0.2370 (13)
C3A	0.8582 (8)	1.0163 (10)	0.1242 (5)	0.046 (2)*	0.2370 (13)
C4A	0.8573 (9)	1.0991 (12)	0.1852 (5)	0.060 (3)*	0.2370 (13)
H4AA	0.7929	1.1638	0.1778	0.072*	0.2370 (13)
H4AB	0.8492	1.0347	0.2180	0.072*	0.2370 (13)
C5A	0.9693 (11)	1.1783 (15)	0.2048 (7)	0.071 (4)*	0.2370 (13)
H5AA	0.9696	1.2288	0.2436	0.086*	0.2370 (13)
C6A	0.9765 (19)	1.2860 (16)	0.1539 (10)	0.048 (6)*	0.2370 (13)
H6AA	0.9095	1.3466	0.1462	0.058*	0.2370 (13)
H6AB	1.0447	1.3436	0.1675	0.058*	0.2370 (13)
C7A	0.9818 (10)	1.2081 (15)	0.0943 (7)	0.061 (4)*	0.2370 (13)
H7AA	0.9876	1.2755	0.0616	0.074*	0.2370 (13)
C8A	0.8709 (10)	1.1192 (12)	0.0728 (5)	0.067 (3)*	0.2370 (13)
H8AA	0.8043	1.1804	0.0628	0.080*	0.2370 (13)
H8AB	0.8742	1.0676	0.0352	0.080*	0.2370 (13)
C9A	1.0848 (12)	1.1107 (16)	0.1064 (7)	0.083 (4)*	0.2370 (13)
H9AA	1.1551	1.1645	0.1203	0.099*	0.2370 (13)
H9AB	1.0884	1.0630	0.0680	0.099*	0.2370 (13)
C10A	0.9617 (16)	0.919 (2)	0.1325 (18)	0.111 (13)*	0.2370 (13)
H10C	0.9623	0.8750	0.0929	0.133*	0.2370 (13)
H10D	0.9560	0.8462	0.1623	0.133*	0.2370 (13)
C11A	1.0752 (17)	1.003 (2)	0.1563 (8)	0.070 (7)*	0.2370 (13)
H11B	1.1415	0.9391	0.1638	0.084*	0.2370 (13)
C12A	1.0707 (12)	1.0799 (15)	0.2168 (6)	0.076 (4)*	0.2370 (13)
H12C	1.0638	1.0127	0.2487	0.091*	0.2370 (13)
H12D	1.1414	1.1324	0.2317	0.091*	0.2370 (13)
C13	0.6623 (2)	0.6114 (3)	−0.01934 (12)	0.0623 (6)	
H13A	0.5809	0.6117	−0.0416	0.075*	0.7630 (13)
C14	0.6717 (2)	0.5413 (2)	0.04345 (11)	0.0569 (6)	
H14A	0.6518	0.4431	0.0367	0.068*	
H14B	0.7510	0.5465	0.0674	0.068*	
C15	0.5944 (2)	0.6070 (2)	0.08101 (12)	0.0579 (6)	
H15A	0.5136	0.6009	0.0580	0.069*	0.2370 (13)
C16	0.6068 (2)	0.5602 (3)	0.14704 (12)	0.0580 (6)	
C17	0.6457 (2)	0.4278 (3)	0.16673 (13)	0.0662 (7)	
H17A	0.6713	0.3675	0.1397	0.079*	
C18	0.6462 (3)	0.3853 (4)	0.22695 (15)	0.0832 (9)	
H18A	0.6705	0.2952	0.2397	0.100*	
C19	0.6121 (3)	0.4727 (5)	0.26767 (15)	0.0919 (10)	
H19A	0.6145	0.4432	0.3082	0.110*	
C20	0.5742 (3)	0.6041 (4)	0.24904 (16)	0.0988 (11)	
H20A	0.5509	0.6642	0.2770	0.119*	
C21	0.5702 (3)	0.6486 (3)	0.18866 (15)	0.0837 (9)	
H21A	0.5429	0.7377	0.1760	0.100*	
C22	0.7301 (2)	0.5415 (2)	−0.06068 (12)	0.0600 (6)	
C23	0.6899 (2)	0.5500 (3)	−0.12483 (13)	0.0745 (8)	
H23A	0.6229	0.6000	−0.1417	0.089*	
C24	0.7487 (3)	0.4843 (4)	−0.16410 (15)	0.0911 (10)	
H24A	0.7208	0.4900	−0.2072	0.109*	
C25	0.8477 (3)	0.4111 (3)	−0.13989 (17)	0.0886 (9)	
H25A	0.8864	0.3663	−0.1664	0.106*	
C26	0.8892 (3)	0.4041 (3)	−0.07700 (17)	0.0907 (10)	
H26A	0.9571	0.3557	−0.0605	0.109*	
C27	0.8312 (3)	0.4684 (3)	−0.03757 (14)	0.0814 (8)	
H27B	0.8606	0.4627	0.0054	0.098*	

### Atomic displacement parameters (Å^2^)

**Table:**

	*U*^11^	*U*^22^	*U*^33^	*U*^12^	*U*^13^	*U*^23^
S1	0.0676 (5)	0.0682 (5)	0.0566 (5)	0.0009 (4)	−0.0002 (4)	0.0033 (4)
O1	0.0770 (15)	0.0821 (16)	0.0802 (16)	0.0303 (14)	0.0244 (13)	0.0188 (14)
N1	0.0547 (13)	0.0405 (12)	0.0571 (14)	−0.0021 (10)	0.0095 (12)	−0.0025 (11)
N2	0.0521 (13)	0.0414 (13)	0.0613 (15)	0.0027 (10)	0.0083 (12)	0.0001 (11)
N3	0.0565 (14)	0.0391 (12)	0.0541 (14)	0.0057 (10)	0.0087 (12)	0.0009 (11)
N4	0.0701 (16)	0.0409 (13)	0.0739 (18)	0.0090 (12)	0.0078 (14)	0.0095 (12)
C1	0.0547 (16)	0.0493 (16)	0.0510 (16)	0.0035 (13)	0.0124 (13)	0.0017 (14)
C2	0.0515 (15)	0.0411 (15)	0.0538 (17)	0.0041 (12)	0.0141 (13)	−0.0011 (12)
C3	0.0520 (16)	0.0426 (16)	0.0537 (18)	0.0045 (12)	0.0100 (14)	0.0000 (13)
C4	0.0642 (19)	0.0578 (18)	0.0619 (19)	−0.0034 (15)	0.0228 (17)	−0.0013 (16)
C5	0.067 (3)	0.065 (2)	0.078 (3)	−0.013 (2)	0.013 (2)	0.0013 (19)
C6	0.099 (5)	0.058 (3)	0.103 (5)	−0.010 (2)	0.008 (3)	−0.017 (2)
C7	0.086 (3)	0.077 (3)	0.058 (2)	0.005 (2)	0.016 (2)	−0.0122 (19)
C8	0.068 (2)	0.063 (2)	0.058 (2)	0.0005 (17)	0.0218 (17)	−0.0007 (16)
C9	0.108 (4)	0.097 (4)	0.060 (2)	−0.011 (3)	0.000 (2)	0.002 (2)
C10	0.056 (2)	0.041 (2)	0.090 (4)	0.0084 (14)	−0.009 (2)	−0.0022 (16)
C11	0.058 (3)	0.065 (3)	0.091 (5)	0.0053 (17)	−0.014 (3)	0.004 (3)
C12	0.062 (2)	0.082 (3)	0.106 (4)	−0.002 (2)	0.005 (2)	−0.013 (3)
C13	0.0650 (14)	0.0609 (14)	0.0593 (14)	0.0091 (12)	0.0114 (12)	−0.0032 (12)
C14	0.0601 (13)	0.0457 (12)	0.0648 (14)	0.0013 (10)	0.0148 (12)	−0.0034 (11)
C15	0.0566 (13)	0.0487 (12)	0.0684 (15)	0.0003 (11)	0.0148 (12)	0.0010 (11)
C16	0.0515 (12)	0.0600 (14)	0.0624 (14)	−0.0037 (11)	0.0133 (11)	−0.0042 (12)
C17	0.0625 (15)	0.0697 (16)	0.0674 (16)	0.0046 (12)	0.0174 (13)	0.0077 (13)
C18	0.0720 (17)	0.096 (2)	0.080 (2)	0.0040 (16)	0.0148 (16)	0.0204 (18)
C19	0.0706 (18)	0.141 (3)	0.0634 (18)	−0.003 (2)	0.0134 (15)	0.005 (2)
C20	0.102 (2)	0.125 (3)	0.074 (2)	−0.002 (2)	0.0295 (19)	−0.026 (2)
C21	0.094 (2)	0.0781 (19)	0.084 (2)	0.0046 (16)	0.0306 (18)	−0.0135 (16)
C22	0.0697 (15)	0.0491 (13)	0.0631 (15)	−0.0009 (11)	0.0192 (13)	−0.0049 (11)
C23	0.0679 (16)	0.088 (2)	0.0664 (17)	−0.0045 (15)	0.0134 (14)	−0.0070 (15)
C24	0.102 (2)	0.110 (3)	0.0661 (18)	−0.016 (2)	0.0285 (18)	−0.0176 (18)
C25	0.117 (3)	0.0713 (19)	0.091 (2)	0.0007 (18)	0.051 (2)	−0.0122 (17)
C26	0.114 (3)	0.0713 (18)	0.098 (2)	0.0306 (18)	0.048 (2)	0.0051 (17)
C27	0.102 (2)	0.0725 (18)	0.0740 (18)	0.0263 (17)	0.0294 (17)	0.0056 (15)

### Geometric parameters (Å, º)

**Table:**

S1—C1	1.668 (3)	C4A—C5A	1.509 (13)
O1—C15	1.223 (3)	C4A—H4AA	0.9700
N1—C1	1.345 (4)	C4A—H4AB	0.9700
N1—N2	1.383 (3)	C5A—C12A	1.507 (15)
N1—C13	1.539 (3)	C5A—C6A	1.54 (2)
N2—C2	1.303 (4)	C5A—H5AA	0.9800
N3—C1	1.370 (4)	C6A—C7A	1.522 (19)
N3—C2	1.373 (4)	C6A—H6AA	0.9700
N3—N4	1.407 (3)	C6A—H6AB	0.9700
N4—H1N4	0.8979	C7A—C9A	1.515 (15)
N4—H2N4	0.8979	C7A—C8A	1.551 (13)
C2—C3	1.493 (4)	C7A—H7AA	0.9800
C3—C10	1.532 (5)	C8A—H8AA	0.9700
C3—C4	1.535 (4)	C8A—H8AB	0.9700
C3—C8	1.539 (4)	C9A—C11A	1.530 (18)
C4—C5	1.522 (5)	C9A—H9AA	0.9700
C4—H4A	0.9700	C9A—H9AB	0.9700
C4—H4B	0.9700	C10A—C11A	1.554 (16)
C5—C12	1.509 (6)	C10A—H10C	0.9700
C5—C6	1.517 (11)	C10A—H10D	0.9700
C5—H5A	0.9800	C11A—C12A	1.532 (18)
C6—C7	1.517 (10)	C11A—H11B	0.9800
C6—H6A	0.9700	C12A—H12C	0.9700
C6—H6B	0.9700	C12A—H12D	0.9700
C7—C9	1.518 (6)	C13—C22	1.509 (3)
C7—C8	1.547 (6)	C13—C14	1.516 (3)
C7—H7A	0.9800	C13—H13A	0.9800
C8—H8A	0.9700	C14—C15	1.513 (3)
C8—H8B	0.9700	C14—H14A	0.9700
C9—C11	1.534 (10)	C14—H14B	0.9700
C9—H9A	0.9700	C15—C16	1.494 (3)
C9—H9B	0.9700	C15—H15A	0.9800
C10—C11	1.542 (6)	C16—C17	1.381 (3)
C10—H10A	0.9700	C16—C21	1.390 (4)
C10—H10B	0.9700	C17—C18	1.384 (4)
C11—C12	1.530 (8)	C17—H17A	0.9300
C11—H11A	0.9800	C18—C19	1.356 (5)
C12—H12A	0.9700	C18—H18A	0.9300
C12—H12B	0.9700	C19—C20	1.364 (5)
S1A—C1A	1.660 (9)	C19—H19A	0.9300
O1A—C13	1.213 (7)	C20—C21	1.385 (5)
N1A—C1A	1.337 (11)	C20—H20A	0.9300
N1A—N2A	1.383 (10)	C21—H21A	0.9300
N1A—C15	1.529 (8)	C22—C23	1.382 (4)
N2A—C2A	1.327 (11)	C22—C27	1.383 (4)
N3A—C2A	1.353 (11)	C23—C24	1.386 (4)
N3A—C1A	1.398 (11)	C23—H23A	0.9300
N3A—N4A	1.409 (11)	C24—C25	1.368 (5)
N4A—H3N4	0.8979	C24—H24A	0.9300
N4A—H4N4	0.8979	C25—C26	1.357 (4)
C2A—C3A	1.519 (13)	C25—H25A	0.9300
C3A—C10A	1.521 (16)	C26—C27	1.375 (4)
C3A—C8A	1.532 (12)	C26—H26A	0.9300
C3A—C4A	1.561 (12)	C27—H27B	0.9300
			
C1—N1—N2	112.8 (2)	C12A—C5A—H5AA	108.5
C1—N1—C13	125.7 (2)	C4A—C5A—H5AA	108.5
N2—N1—C13	120.7 (2)	C6A—C5A—H5AA	108.5
C2—N2—N1	105.1 (2)	C7A—C6A—C5A	108.8 (11)
C1—N3—C2	109.7 (2)	C7A—C6A—H6AA	109.9
C1—N3—N4	124.6 (2)	C5A—C6A—H6AA	109.9
C2—N3—N4	125.7 (2)	C7A—C6A—H6AB	109.9
N3—N4—H1N4	95.2	C5A—C6A—H6AB	109.9
N3—N4—H2N4	122.7	H6AA—C6A—H6AB	108.3
H1N4—N4—H2N4	121.9	C9A—C7A—C6A	110.2 (12)
N1—C1—N3	102.9 (2)	C9A—C7A—C8A	108.5 (11)
N1—C1—S1	130.5 (2)	C6A—C7A—C8A	109.0 (12)
N3—C1—S1	126.6 (2)	C9A—C7A—H7AA	109.7
N2—C2—N3	109.6 (3)	C6A—C7A—H7AA	109.7
N2—C2—C3	124.4 (3)	C8A—C7A—H7AA	109.7
N3—C2—C3	126.0 (2)	C3A—C8A—C7A	110.5 (9)
C2—C3—C10	109.4 (3)	C3A—C8A—H8AA	109.5
C2—C3—C4	112.3 (3)	C7A—C8A—H8AA	109.5
C10—C3—C4	109.9 (4)	C3A—C8A—H8AB	109.5
C2—C3—C8	109.9 (2)	C7A—C8A—H8AB	109.5
C10—C3—C8	106.6 (5)	H8AA—C8A—H8AB	108.1
C4—C3—C8	108.7 (2)	C7A—C9A—C11A	110.2 (12)
C5—C4—C3	110.7 (3)	C7A—C9A—H9AA	109.6
C5—C4—H4A	109.5	C11A—C9A—H9AA	109.6
C3—C4—H4A	109.5	C7A—C9A—H9AB	109.6
C5—C4—H4B	109.5	C11A—C9A—H9AB	109.6
C3—C4—H4B	109.5	H9AA—C9A—H9AB	108.1
H4A—C4—H4B	108.1	C3A—C10A—C11A	110.3 (14)
C12—C5—C6	110.5 (5)	C3A—C10A—H10C	109.6
C12—C5—C4	109.9 (3)	C11A—C10A—H10C	109.6
C6—C5—C4	108.2 (5)	C3A—C10A—H10D	109.6
C12—C5—H5A	109.4	C11A—C10A—H10D	109.6
C6—C5—H5A	109.4	H10C—C10A—H10D	108.1
C4—C5—H5A	109.4	C9A—C11A—C12A	108.9 (13)
C5—C6—C7	110.6 (4)	C9A—C11A—C10A	108.1 (17)
C5—C6—H6A	109.5	C12A—C11A—C10A	109.5 (18)
C7—C6—H6A	109.5	C9A—C11A—H11B	110.1
C5—C6—H6B	109.5	C12A—C11A—H11B	110.1
C7—C6—H6B	109.5	C10A—C11A—H11B	110.1
H6A—C6—H6B	108.1	C5A—C12A—C11A	109.8 (12)
C6—C7—C9	109.4 (5)	C5A—C12A—H12C	109.7
C6—C7—C8	109.1 (4)	C11A—C12A—H12C	109.7
C9—C7—C8	108.8 (3)	C5A—C12A—H12D	109.7
C6—C7—H7A	109.8	C11A—C12A—H12D	109.7
C9—C7—H7A	109.8	H12C—C12A—H12D	108.2
C8—C7—H7A	109.8	O1A—C13—C22	121.9 (4)
C3—C8—C7	110.3 (3)	O1A—C13—C14	112.7 (4)
C3—C8—H8A	109.6	C22—C13—C14	114.8 (2)
C7—C8—H8A	109.6	O1A—C13—N1	84.7 (4)
C3—C8—H8B	109.6	C22—C13—N1	110.0 (2)
C7—C8—H8B	109.6	C14—C13—N1	107.8 (2)
H8A—C8—H8B	108.1	O1A—C13—H13A	23.7
C7—C9—C11	109.7 (4)	C22—C13—H13A	108.0
C7—C9—H9A	109.7	C14—C13—H13A	108.0
C11—C9—H9A	109.7	N1—C13—H13A	108.0
C7—C9—H9B	109.7	C15—C14—C13	113.0 (2)
C11—C9—H9B	109.7	C15—C14—H14A	109.0
H9A—C9—H9B	108.2	C13—C14—H14A	109.0
C3—C10—C11	110.5 (3)	C15—C14—H14B	109.0
C3—C10—H10A	109.5	C13—C14—H14B	109.0
C11—C10—H10A	109.5	H14A—C14—H14B	107.8
C3—C10—H10B	109.5	O1—C15—C16	121.9 (2)
C11—C10—H10B	109.5	O1—C15—C14	119.7 (2)
H10A—C10—H10B	108.1	C16—C15—C14	118.3 (2)
C12—C11—C9	109.5 (4)	O1—C15—N1A	66.7 (3)
C12—C11—C10	109.6 (7)	C16—C15—N1A	102.4 (3)
C9—C11—C10	108.5 (5)	C14—C15—N1A	105.0 (3)
C12—C11—H11A	109.7	O1—C15—H15A	43.6
C9—C11—H11A	109.7	C16—C15—H15A	110.2
C10—C11—H11A	109.7	C14—C15—H15A	110.2
C5—C12—C11	109.6 (3)	N1A—C15—H15A	110.2
C5—C12—H12A	109.7	C17—C16—C21	119.0 (3)
C11—C12—H12A	109.7	C17—C16—C15	121.9 (2)
C5—C12—H12B	109.7	C21—C16—C15	119.0 (2)
C11—C12—H12B	109.7	C16—C17—C18	119.6 (3)
H12A—C12—H12B	108.2	C16—C17—H17A	120.2
C1A—N1A—N2A	115.4 (7)	C18—C17—H17A	120.2
C1A—N1A—C15	124.4 (7)	C19—C18—C17	121.2 (3)
N2A—N1A—C15	120.2 (6)	C19—C18—H18A	119.4
C2A—N2A—N1A	102.8 (7)	C17—C18—H18A	119.4
C2A—N3A—C1A	109.9 (7)	C18—C19—C20	119.9 (3)
C2A—N3A—N4A	126.3 (8)	C18—C19—H19A	120.1
C1A—N3A—N4A	123.7 (7)	C20—C19—H19A	120.1
N3A—N4A—H3N4	118.9	C19—C20—C21	120.3 (3)
N3A—N4A—H4N4	112.9	C19—C20—H20A	119.8
H3N4—N4A—H4N4	84.7	C21—C20—H20A	119.8
N1A—C1A—N3A	101.2 (7)	C20—C21—C16	120.0 (3)
N1A—C1A—S1A	131.6 (7)	C20—C21—H21A	120.0
N3A—C1A—S1A	127.1 (7)	C16—C21—H21A	120.0
N2A—C2A—N3A	110.7 (8)	C23—C22—C27	118.0 (3)
N2A—C2A—C3A	121.1 (8)	C23—C22—C13	118.9 (2)
N3A—C2A—C3A	128.2 (8)	C27—C22—C13	123.2 (2)
C2A—C3A—C10A	110.3 (11)	C22—C23—C24	120.3 (3)
C2A—C3A—C8A	112.6 (8)	C22—C23—H23A	119.8
C10A—C3A—C8A	105.1 (16)	C24—C23—H23A	119.8
C2A—C3A—C4A	107.4 (9)	C25—C24—C23	120.4 (3)
C10A—C3A—C4A	112.0 (14)	C25—C24—H24A	119.8
C8A—C3A—C4A	109.5 (8)	C23—C24—H24A	119.8
C5A—C4A—C3A	108.3 (9)	C26—C25—C24	119.8 (3)
C5A—C4A—H4AA	110.0	C26—C25—H25A	120.1
C3A—C4A—H4AA	110.0	C24—C25—H25A	120.1
C5A—C4A—H4AB	110.0	C25—C26—C27	120.3 (3)
C3A—C4A—H4AB	110.0	C25—C26—H26A	119.9
H4AA—C4A—H4AB	108.4	C27—C26—H26A	119.9
C12A—C5A—C4A	111.1 (11)	C26—C27—C22	121.2 (3)
C12A—C5A—C6A	111.3 (12)	C26—C27—H27B	119.4
C4A—C5A—C6A	109.0 (12)	C22—C27—H27B	119.4
			
C1—N1—N2—C2	−0.2 (3)	C10A—C3A—C4A—C5A	56.4 (16)
C13—N1—N2—C2	−170.9 (2)	C8A—C3A—C4A—C5A	−59.8 (11)
N2—N1—C1—N3	0.5 (3)	C3A—C4A—C5A—C12A	−59.7 (13)
C13—N1—C1—N3	170.5 (2)	C3A—C4A—C5A—C6A	63.2 (13)
N2—N1—C1—S1	179.8 (2)	C12A—C5A—C6A—C7A	58.1 (17)
C13—N1—C1—S1	−10.1 (5)	C4A—C5A—C6A—C7A	−64.7 (16)
C2—N3—C1—N1	−0.5 (3)	C5A—C6A—C7A—C9A	−58.3 (17)
N4—N3—C1—N1	−178.1 (3)	C5A—C6A—C7A—C8A	60.7 (16)
C2—N3—C1—S1	−179.9 (2)	C2A—C3A—C8A—C7A	176.5 (10)
N4—N3—C1—S1	2.5 (4)	C10A—C3A—C8A—C7A	−63.3 (14)
N1—N2—C2—N3	−0.1 (3)	C4A—C3A—C8A—C7A	57.1 (12)
N1—N2—C2—C3	178.8 (3)	C9A—C7A—C8A—C3A	62.0 (13)
C1—N3—C2—N2	0.4 (3)	C6A—C7A—C8A—C3A	−58.0 (14)
N4—N3—C2—N2	178.0 (3)	C6A—C7A—C9A—C11A	60.5 (16)
C1—N3—C2—C3	−178.5 (3)	C8A—C7A—C9A—C11A	−58.8 (15)
N4—N3—C2—C3	−0.9 (5)	C2A—C3A—C10A—C11A	−174.6 (18)
N2—C2—C3—C10	3.2 (6)	C8A—C3A—C10A—C11A	64 (2)
N3—C2—C3—C10	−178.1 (5)	C4A—C3A—C10A—C11A	−55 (3)
N2—C2—C3—C4	125.5 (3)	C7A—C9A—C11A—C12A	−59.8 (17)
N3—C2—C3—C4	−55.8 (4)	C7A—C9A—C11A—C10A	59.1 (18)
N2—C2—C3—C8	−113.4 (3)	C3A—C10A—C11A—C9A	−63 (3)
N3—C2—C3—C8	65.3 (4)	C3A—C10A—C11A—C12A	56 (3)
C2—C3—C4—C5	−178.8 (3)	C4A—C5A—C12A—C11A	62.8 (15)
C10—C3—C4—C5	−56.8 (5)	C6A—C5A—C12A—C11A	−58.8 (16)
C8—C3—C4—C5	59.5 (4)	C9A—C11A—C12A—C5A	58.7 (17)
C3—C4—C5—C12	59.5 (4)	C10A—C11A—C12A—C5A	−59.2 (17)
C3—C4—C5—C6	−61.2 (5)	C1—N1—C13—O1A	−14.9 (5)
C12—C5—C6—C7	−58.6 (7)	N2—N1—C13—O1A	154.5 (4)
C4—C5—C6—C7	61.7 (7)	C1—N1—C13—C22	107.2 (3)
C5—C6—C7—C9	58.6 (7)	N2—N1—C13—C22	−83.5 (3)
C5—C6—C7—C8	−60.3 (7)	C1—N1—C13—C14	−127.0 (3)
C2—C3—C8—C7	179.5 (3)	N2—N1—C13—C14	42.3 (3)
C10—C3—C8—C7	61.1 (4)	O1A—C13—C14—C15	−29.6 (5)
C4—C3—C8—C7	−57.3 (4)	C22—C13—C14—C15	−175.0 (2)
C6—C7—C8—C3	58.0 (5)	N1—C13—C14—C15	62.1 (3)
C9—C7—C8—C3	−61.3 (4)	C13—C14—C15—O1	12.7 (3)
C6—C7—C9—C11	−59.2 (6)	C13—C14—C15—C16	−172.1 (2)
C8—C7—C9—C11	59.9 (5)	C13—C14—C15—N1A	−58.7 (4)
C2—C3—C10—C11	179.7 (6)	C1A—N1A—C15—O1	18.2 (7)
C4—C3—C10—C11	56.0 (8)	N2A—N1A—C15—O1	−160.4 (8)
C8—C3—C10—C11	−61.6 (8)	C1A—N1A—C15—C16	−101.5 (8)
C7—C9—C11—C12	59.6 (5)	N2A—N1A—C15—C16	80.0 (7)
C7—C9—C11—C10	−60.0 (6)	C1A—N1A—C15—C14	134.4 (8)
C3—C10—C11—C12	−57.8 (8)	N2A—N1A—C15—C14	−44.1 (7)
C3—C10—C11—C9	61.7 (8)	O1—C15—C16—C17	147.4 (3)
C6—C5—C12—C11	58.4 (6)	C14—C15—C16—C17	−27.7 (3)
C4—C5—C12—C11	−60.9 (5)	N1A—C15—C16—C17	−142.5 (4)
C9—C11—C12—C5	−58.9 (5)	O1—C15—C16—C21	−27.7 (4)
C10—C11—C12—C5	60.0 (6)	C14—C15—C16—C21	157.1 (2)
C1A—N1A—N2A—C2A	0.7 (10)	N1A—C15—C16—C21	42.4 (4)
C15—N1A—N2A—C2A	179.4 (7)	C21—C16—C17—C18	0.7 (4)
N2A—N1A—C1A—N3A	−1.4 (10)	C15—C16—C17—C18	−174.5 (2)
C15—N1A—C1A—N3A	179.9 (6)	C16—C17—C18—C19	−1.7 (4)
N2A—N1A—C1A—S1A	−177.2 (7)	C17—C18—C19—C20	1.3 (5)
C15—N1A—C1A—S1A	4.2 (14)	C18—C19—C20—C21	0.1 (5)
C2A—N3A—C1A—N1A	1.6 (10)	C19—C20—C21—C16	−1.2 (5)
N4A—N3A—C1A—N1A	179.1 (8)	C17—C16—C21—C20	0.7 (4)
C2A—N3A—C1A—S1A	177.6 (7)	C15—C16—C21—C20	176.0 (3)
N4A—N3A—C1A—S1A	−4.9 (13)	O1A—C13—C22—C23	8.0 (6)
N1A—N2A—C2A—N3A	0.4 (10)	C14—C13—C22—C23	149.9 (2)
N1A—N2A—C2A—C3A	178.7 (8)	N1—C13—C22—C23	−88.4 (3)
C1A—N3A—C2A—N2A	−1.3 (11)	O1A—C13—C22—C27	−171.8 (5)
N4A—N3A—C2A—N2A	−178.7 (8)	C14—C13—C22—C27	−30.0 (4)
C1A—N3A—C2A—C3A	−179.5 (9)	N1—C13—C22—C27	91.8 (3)
N4A—N3A—C2A—C3A	3.1 (15)	C27—C22—C23—C24	1.3 (4)
N2A—C2A—C3A—C10A	4 (2)	C13—C22—C23—C24	−178.6 (3)
N3A—C2A—C3A—C10A	−177.6 (18)	C22—C23—C24—C25	−0.4 (5)
N2A—C2A—C3A—C8A	121.5 (10)	C23—C24—C25—C26	−0.8 (5)
N3A—C2A—C3A—C8A	−60.5 (14)	C24—C25—C26—C27	1.0 (5)
N2A—C2A—C3A—C4A	−118.0 (10)	C25—C26—C27—C22	−0.1 (5)
N3A—C2A—C3A—C4A	60.1 (12)	C23—C22—C27—C26	−1.0 (4)
C2A—C3A—C4A—C5A	177.7 (9)	C13—C22—C27—C26	178.8 (3)

### Hydrogen-bond geometry (Å, º)

**Table:**

*D*—H···*A*	*D*—H	H···*A*	*D*···*A*	*D*—H···*A*
N4—H1*N*4···S1^i^	0.90	2.60	3.475 (3)	166
C4—H4*B*···N4	0.97	2.53	3.177 (4)	124

Symmetry code: (i) −*x*+1, −*y*+2, −*z*.
